# The Valued Life Activities Scale (VLAs): linguistic validation, cultural adaptation and psychometric testing in people with rheumatic and musculoskeletal diseases in the UK

**DOI:** 10.1186/s12891-020-03409-9

**Published:** 2020-07-30

**Authors:** Y. Prior, A. Tennant, S. Tyson, A. Hammond

**Affiliations:** 1 Centre for Health Sciences Research, University of Salford, Frederick Road Campus, Brian Blatchford Building PO.50, Salford, M6 6PU UK; 2 Leeds Institute of Rheumatic and Musculoskeletal Medicine, University of Leeds, Leeds, UK; 3 Division of Nursing, Midwifery and Social Work, University of Manchester, Manchester, UK

**Keywords:** RMDs, PROMS, Participation, Activities, Leisure, Activities of daily living, Valued life activities, Rasch analysis, Validity, Reliability, RMDs (rheumatic and musculoskeletal diseases), PROMS (patient reported outcome measures)

## Abstract

**Background:**

The Valued Life Activities Scale (VLAs) measures difficulty in daily activities and social participation. With various versions involving a different number of items, we have linguistically and culturally adopted the full VLAs (33-items) and psychometrically tested it in adults with rheumatic and musculoskeletal diseases in the United Kingdom.

**Methods:**

Participants with Rheumatoid Arthritis, Ankylosing Spondylitis, Chronic Pain/ Fibromyalgia, Chronic Hand/ Upper Limb Conditions, Osteoarthritis, Systemic Lupus, Systemic Sclerosis and Primary Sjogren’s Syndrome were recruited from out-patient clinics in National Health Service Hospitals, General Practice and patient organisations in the UK. Phase1 involved linguistic and cultural adaptation: forward translation to British English; synthesis; expert panel review and cognitive debriefing interviews. In Phase2 participants completed postal questionnaires to assess internal construct validity using (i) Confirmatory Factor Analysis (CFA) (ii) Mokken scaling and (iii) Rasch model.

**Results:**

Responders (*n* = 1544) had mean age of 59 years (SD13.3) and 77.2% women. A CFA failed to support a total score from the 33-items (Chi Square 3552:df 464: *p* < 0.0001). Mokken scaling indicated a strong non-parametric association between items. Fit to the Rasch model indicated that the VLAs was characterised by multidimensionality and item misfit, which may have been influenced by clusters of residual item correlations. An item banking approach resolved a 25-item calibrated set whose application could accommodate the ‘does not apply to me’ response option.

**Conclusions:**

The UK version of the VLAs failed to satisfy classical and modern psychometric standards for complete item sets. However, as the scale is not usually applied in complete format, an item bank approach calibrated 25 items with fit to the Rasch model. Suitable Computer Adaptive Testing (CAT) software could implement the item set, giving patients the choice of whether an item applies to them, or not.

## Background

Rheumatic and musculoskeletal diseases (RMDs) such as Osteoarthritis (OA), Rheumatoid Arthritis (RA), Chronic Pain (CP) and Fibromyalgia (FM), are common, and their prevalence is rising with the ageing population [1]. Many individuals with RMDs report moderate to high pain and fatigue which can lead to activity limitation and participation restriction, which affect Quality of Life (QoL) [2–5]. Therefore, European League Against Rheumatism (EULAR) recommendations for health professionals’ approach to pain management in inflammatory arthritis and OA, emphasise pain is a complex and multifaceted experience. Treatment should be guided by patient’s preferences and priorities, such as the impact on their activities and participation, in order to facilitate improved health outcomes [6]. Patient reported outcome measures (PROMs) can be used to identify such preferences and priorities. However, few include both activities and participation items.

Developed in the United States (USA), the Valued Life Activities scale (VLAs) is one such PROM, measuring both difficulty in daily activities and participation in society [7]. It was developed from the 75-item Activities Enumeration Index [8] which was derived from content analysis of diaries and telephone interviews with patients with RA or OA [7, 9–11].

The VLAs is based on Verbrugge and Jette’s disablement model [12]. This defines activity and participation in three domains:

- Obligatory: required for survival and self-sufficiency, such as eating, hygiene, walking and transport
- Committed: related to one’s principal social roles, such as paid work, child and family care and household responsibilities and
- Discretionary: engaged in for relaxation and pleasure, such as socialising, exercise, leisure, hobbies, religious activities, travel, volunteer work, educational activities, gardening.

The VLAs developers have allocated items to these three domains based on the model’s definitions [7] (Additional File 1).

The VLAs has been used in over 10 cohort studies with large numbers of people with RA and systemic lupus erythematosus (SLE), but the way in which it has been administered varies, with different studies using different numbers of items (i.e. 33, 29, 26, 21, or 14 items) – see Additional Table 1); some items differing between versions (depending on diagnosis), and several different scoring methods being used. These methods include: the average difficulty score for all items and for each of the three domains; the average score created by adjusting scores if the person reports changing how they perform the activity (e.g. use an assistive device, have help, take more time or limit time performing), with item scores being increased by one point if the score < 2 [13]; or calculating (unadjusted) scores only for those items identified as important by the participant [7, 14]. Accordingly, we requested the definitive version and scoring method from the lead scale developer (Dr P. Katz). This was identified as the 33-item version scored on a 4-point scale (0 = no difficulty to 3 = unable to do). People are asked to record for each item: whether it is not applicable to them (i.e. the person does not normally perform the activity for reasons unrelated to their condition); their degree of difficulty performing it; and whether the item is important to them [13]. The overall score is then calculated as the mean of only those items identified as both applicable and important. As a result, different respondents’ scores are based on different numbers of items within the VLAs, as the intention is to score only those activities which are “valued” by participants.

Some psychometric testing has been conducted with the 33-item and shorter versions, although with differing scoring methods, demonstrating internal consistency and test-retest reliability. The 14-item Short-VLAs, was developed using Rasch analysis, and unidimensionality, construct and concurrent validity have also been demonstrated [15, 16]. However, the variability in how the tool has been administered (differing numbers of items) and scoring methods means there is currently limited evidence for the reliability and validity of the 33-item VLAs.

The VLAs, and the way in which is used, presents considerable challenges to deliver a robust psychometric analysis. For example, in the full 33-item set, respondents may simply respond to an item saying ‘it is not relevant to me’ then, in practice, a valid response may arise from any combination of the 33 items. As such, there are a vast number of possible combinations available (33 factorial). The current practice is to average the responses to the chosen items, giving a total score in the range 0–3. There are two major problems with this approach; the responses are ordinal and do not support mathematical operations such as averaging, which requires at least interval scaling. Even if this is unfortunately ignored, such averaging would only be interpretable if every item had the same level of difficulty. Neither of these conditions hold for items in ordinal scales [17, 18].

How then can a scale such as the VLAs be shown to be psychometrically sound? To satisfy traditional psychometric standards, the various items sets need to be shown to be reliable, valid, unidimensional and invariant for key groups (32). The items themselves need to be locally independent (conditional on the trait), although failure of this requirement often reflects a degree of item redundancy. The key issue here is that the item set, from which choices of relevant items are made, is robust from a psychometric perspective.

Nevertheless, even if the various versions of the scale are shown to be robust, there remains the challenge of the scoring associated with, potentially, a very large number of subsets as chosen by the user. It is here that a variation of Computer Adaptive Testing (CAT) can resolve the issue. With a calibrated set of items (e.g. indicating the level of difficulty associated with each of the 33 VLAs items), these can be administered to the respondent, as long as there is a ‘not relevant for me’ option, which will be treated as a missing value by the CAT, so moving on to the next item.

Consequently, the analytical strategy required is to first assess the traditional psychometric properties of the VLAs versions, and then proceed to determine if a calibrated item set suitable for CAT can be found, given any limitations observed in the traditional analysis.

Before a PROM can be used in another language, or country with the same language, it is necessary to adapt the PROM and psychometrically test it in the target group(s). Thus the aims of this study were to develop a British English version of the VLAs (using the full 33-item scale) following recommended linguistic and cultural adaptation guidelines [19, 20], and to test its psychometric properties in adults with RMDs in the United Kingdom (UK). We also investigated the psychometric properties of two shorter versions of the VLAs (26 and 14-items), embedded within the 33-item definitive version. The 26-item version, which had split the ‘physical activities’ item into two, but was included as one item, as in the 33-item version, so making it, in practice, a 25-item version, together with the 14-item short form. Thus, the adaptation, and following psychometric analysis focused on the 33, 26(25) and 14-item versions.

## Method

### Study setting

Recruitment of people with RA was conducted through rheumatology outpatient clinics in 17 National Health Service (NHS) Hospitals. Participants with RA from a previous PROM study were also contacted [21]. Recruitment of people with the other seven RMDs was from 19 rheumatology or orthopaedic out-patient hospital departments, four General Practitioner (GP) surgeries, and from 10 RMD patient organisations in the UK.

### Eligibility criteria

Inclusion criteria were people: aged ≥18 years; diagnosed with Rheumatoid Arthritis (RA), Ankylosing Spondylitis (AS), Chronic Pain (CP) or Fibromyalgia (FM), Chronic Hand and Upper Limb Conditions (CHUL), Osteoarthritis (OA), Systemic Lupus (SLE), Systemic Sclerosis (SS), and Primary Sjogren’s Syndrome (PSS) by either a rheumatology consultant, or an orthopaedic consultant, GP or extended-scope health professional (in the case of OA and CP/FM specifically); able to read, write and understand English; and provide written informed consent.

### Procedures

#### Phase-1: cross-cultural adaptation

We followed recommendations for linguistic and cross-cultural adaptation [19, 20]. As the 33-item VLAs is written in North American English, backward translation was not required [Additional File 1]. Two native British English speakers forward translated the VLAs; one of whom was a rheumatology occupational therapist and the other was not involved in health care and was unfamiliar with health outcome measures. Following forward translation, the two translators resolved any discrepancies. A North American speaker, with an academic background, also helped with checking that the forward translation reflected the accurate meaning of the item sets. An Expert Panel, consisting of three occupational therapists, a physiotherapist, a methodologist and a layperson with RA (all English speakers as their first language) discussed the translation to agree a prototype British English VLAs. This was then reviewed by the panel for semantic (i.e. do words mean the same thing), idiomatic (e.g. presence of colloquialism or idioms), experiential and conceptual equivalence to the original 33-item North American English version of the VLAs.

### Cognitive de-briefing interviews

Cognitive de-briefing interviews were conducted with a purposive sample of participants with RA identified from the participants of a previous study residing within the Midlands and North West of England [21]. The sample included a wide range of demographic characteristics and health status (i.e. range of age, gender, disease duration and work status). The questionnaire booklet was posted for completion at home one week before a cognitive de-briefing interview conducted face-to-face or by telephone by an occupational therapist, depending on the participant’s preference.

These semi-structured interviews determined whether the VLAs items were relevant, understandable and comprehensive, and to confirm participants’ understanding of the items matches the intended use [19]. Participants were asked to rate the relevance and comprehensibility of the VLAs using a five-point likert scale (1 = not relevant to 5 = very relevant; and 1 = very easy to understand to 5 = very difficult to understand). Interviews were audio-recorded and transcribed for ease of content analyses. A preliminary report of the findings was reviewed by the Expert Panel to agree on recommended changes prior to finalisation. A final version of this report and the British English VLAs were submitted to the lead developer in the USA for review and the lead developer approved the changes.

#### Phase-2: psychometric testing

##### Participants

**Fig. 1:**
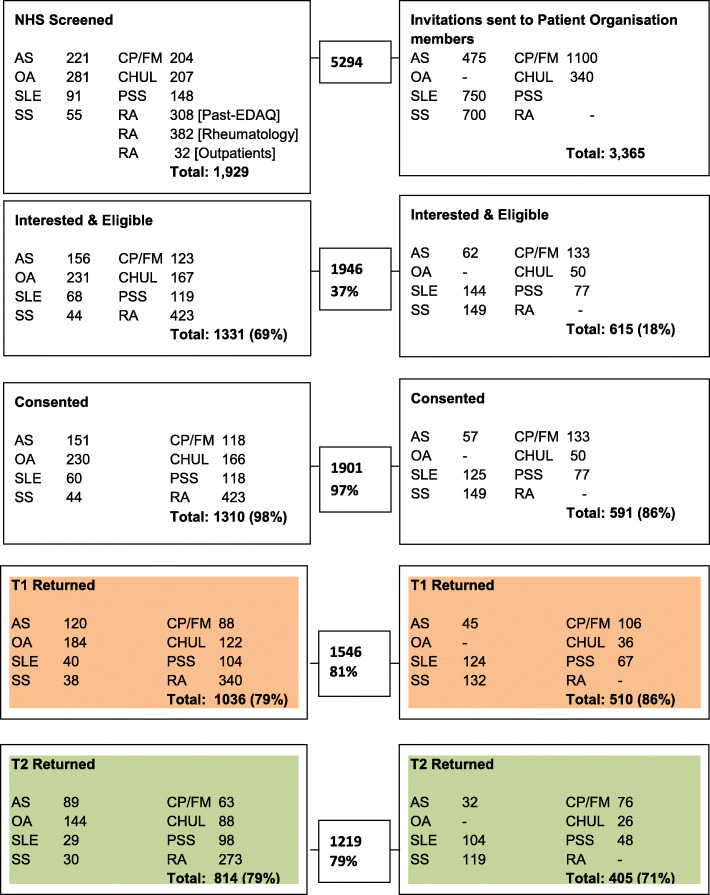
British VLAs Overall Recruitment & Study Progress Flow Diagram

Participants with one of the eight RMDs as their primary diagnosis were recruited by research nurses or therapists using an eligibility checklist to screen patients. Additionally, patient organisations, such as the National Rheumatoid Arthritis Society (NRAS), Arthritis Care, National Ankylosing Spondylitis Society (NASS) and Fibromyalgia Action UK (FMA UK), mailed out study invitation letters, information sheets and a reply form to random samples of their members to help recruit participants. The reply form included the eligibility checklist items. Both rural and urban populations and a wide mix of socio-demographic characteristics were included (Fig. 1).

##### Data collection

Data were collected using postal questionnaires. The questionnaire booklet included demographic and health data (e.g. age, gender, marital, educational and employment status, disease duration, medication regimen), the 33-item VLAs and two measures of physical function: the Health Assessment Questionnaire (HAQ) [22], the SF36 v2.0 [23]; as well as a 0–10 Numeric Rating Scale (NRS) reporting disease activity.

### Sample size

The sample size calculation for Rasch analysis suggested that a sample of at least 150 for each condition will give 99% confidence of the person estimate being within ±0.5 logits, irrespective of whether or not the scale is well targeted to the patients [24]. We chose to recruit a higher number of people with RA as we aimed to conduct secondary analysis with the RA data, if the VLAs demonstrated appropriate psychometric properties. We stopped recruitment once we had at least 150 sufficiently completed questionnaire booklets.

### Statistical analysis

#### Confirmatory factor analysis

The VLAs has undergone revision over time, such that there are several versions with 33- items being the definitive version. The other versions are nested within the 33-item scale, but the 26-item version includes two items for physical recreational activities (moderate and vigorous), rather than one item, as in the 33-item VLAs. Accordingly, when testing two shorter versions of the VLAs, we derived a 25-item VLAs (rather than 26- item version) from the 33-item version, as well as testing the Short VLAs (SVLAs: 14 items).

Confirmation of the 33-item structure from a classical test perspective would follow from a Confirmatory Factor Analysis (CFA) where a priori there is evidence that the item set constitutes one, or a series of domains [25]. Following Kline, fit is determined by a non-significant chi square statistic [26]. Ancillary fit statistics include the RMSEA where a value less than 0.06 would be appropriate, the Comparative Fit Index (CFI), a comparison of final model and baseline model, and the Tucker Lewis Index (TLI), another incremental fit Index which adds penalties for increasing the parameters. Both indices would suggest good fit with values above 0.95. Thus, in the present study, the item set is fit to a CFA model in Mplus [27] and tested for the three domains (Obligatory, Committed and Discretionary) and the total score only for “important and applicable” items.

#### Mokken scaling

The Mokken scale is a non-parametric probabilistic model that utilises the Loevingers H coefficient to determine the ‘scalability’ of a set of items. H appears to be a measure of the degree to which the score is able to discriminate between persons in the given sample [28]. It has been argued that Mokken scaling is a natural starting point for item analysis, and it is used here in that context, to identify if any items from the VLAs display a level of discrimination inconsistent with the expectations of the Rasch model, as represented by low values (< 0.3) of H [29]. In the present study Mokken scaling is examined through the *msp* procedure in STATA 13 [30].

#### Rasch model

Data from the 33 items were fitted to the Rasch model to ascertain if a quantitative structure was present within the domain(s) being measured [31]. Described in detail elsewhere [32], the process is used to test fit to the model expectations, unidimensionality, (conditional) local item independence and invariance (Differential Item Functioning) by contextual groups of age, gender, employment and marital status, duration of disease, and where data are pooled, by condition [33, 34]. Briefly, the RUMM2030 Rasch software [35] has a summary Chi-Square Interaction statistic, which should be above 0.05 if data fit the model. It has residual item and person means and standard deviations, the latter which need to below 1.4 to ensure no individual item is beyond a ± 2.5 range. Reliability of the items set was also reported in the form of a ‘person separation Index’ which, should the data have a normal distribution, is equivalent to Cronbach’s Alpha (internal consistency) [36], else the value will deviate from Alpha. A post hoc t-test is undertaken to determine unidimensionality, contrasting two estimates derived from item sub-sets loading positive and negative on the first residual principal component [37]. The number of contrasts between estimates where the t-test < 0.05 should not exceed 5% to be indicative of unidimensionality (or the lower confidence interval of that proportion of tests).

Following this, informed by the above analysis of 33 items, a calibration of the item set was attempted to form the basis of a CAT. To avoid the potential bias caused by a breach of the local independence assumption, first a set of ‘core’ items that fit the model and were free of local dependency were identified [38]. In doing so surplus items were set aside into a series of secondary item sets, which were subsequently fit to the model, anchored to the core metric by items in the core set which were free of dependency. Fit of the core and subsequent item sets to the Rasch model were tested by repeated sampling of the total data set to ensure the Type 1 error rate of the fit is accurate [39]. In this way, a calibrated set of items became available that could be administered in an innovative fashion by appropriate CAT software. The efficacy of the CAT process was evaluated by simulation using the Firestar programme [40].

The analysis uses the RUMM2030 software utilising the partial credit parameterisation of the Rasch model [35, 41].

## Results

### Phase 1: cross-cultural adaptation

Cognitive debriefing interviews were conducted with 31 participants with RA whose socio-demographic and health characteristics are detailed in Table 1.

**Table 1:** The British VLAs Socio-demographic Characteristics of Participants [Phase 2 – T^1^]

	AS (*n* = 165)	OA (*n* = 184)	SLE (*n* = 164)	SS (*n* = 170)	CP/FM (*n* = 194)	CH/UL (*n* = 158)	PSS (*n* = 171)	RA (*n* = 340)
Response Rate (%)	79	80	89	88	77	73	88	80
Age (Mean (SD))	53.48 (13.89)	63.28 (10.41)	53.20 (13.22)	65.52 (10.65)	53.10 (13.47)	54.66 (14.13)	62.88 (11.25)	61.96 (12.09)
Gender (M: F)	118:45	41:143	7:157	12:158	24:170	48:109	10:160	89:251
Recruited from: NHS: Patient Organisation	120:45	184:0	40:124	38:132	88:106	122:36	104:67	
Condition duration in years (Mean (SD):	24.75 (15.16)	10.43 (10.24)	18.22 (11.54)	16.67 (11.15)	12.96 11.62)	6.0 (12.34)	16.44 (17.04)	14.44 (11.73)
Marital status: n (%):								
Single/Divorced/Widowed	38 (33%)	46 (25%)	31 (19%)	58 (34%)	58 (30%)	42 (26%)	54 (32%)	94 (28%)
Married/Living with partner	126 (77%)	136 (74%)	133 (81%)	111 (66%)	136 (70%)	115 (74%)	117 (68%)	241 (71%)
Missing	1 (0.0%)	2 (0.0%)	0	1 (0.0%)	0	1 (0.0%)	0	5 (1%)
Living status: n (%)								
Alone	26 (16%)	43 (23%)	17 (10%)	52 (30%)	38(20%)	36 (24%)	47 (28%)	70 (21%)
Family/Significant other	134 (81%)	133 (73%)	143 (87%)	115 (68%)	145 (75%)	113 (71%)	121 (71%)	245 (72%)
Missing	5 (3%)	8 (4%)	9 (3%)	3 (2%)	11 (5%)	8 (5%)	3 (1%)	25 (7%)
Children living at home n (%):	41 (25%)	12 (7%)	28 (17%)	3 (2%)	47 (24%)	24 (15%)	17 (10%)	36 (10.5)
Employment status:								
Paid employment	87 (53%)	56 (30%)	62 (38%)	38 (22%)	70 (36%)	80 (51%)	64 (38%)	108 (32%)
Long term sick leave	7 (4%)	5 (3%)	9 (6%)	2 (1%)	14 (7%)	10 (6%)	2 (1%)	9 (2%)
Early retired ill-health	19 (12%)	13 (7%)	31 (19%)	29 (17%)	30 (16%)	10 (6%)	16 (10%)	35 (10%)
Unemployed	5 (3%)	3 (2%)	1 (1%)	1 (1%)	16 (8%)	4 (3%)	2 (1%)	4 (1%)
Retired	41 (25%)	103 (57%)	44 (26%)	95 (56%)	38 (26%)	44 (28%)	82 (48%)	169 (50%)
Homemaker	5 (3%)	2 (1%)	14 (9%)	4 (2%)	21 (11%)	6 (4%)	3 (2%)	8 (2%)
Student	0	0	2 (1%)	0	3 (1%)	1 (0.0%)	0	1 (0.0%)
Missing	1 (0.0%)	2 (0.0%)	1 (0.0%)	1 (0.0%)	2 (0.0%)	3 (0.0%)	1 (0.0%)	5 (1%)
Education level (ISCED):								
Level 1: Compulsory school	19 (12%)	47 (25%)	16 (10%)	23 (13%)	20 (10%)	23 (15%)	22 (13%)	78 (23%)
Level 2: Secondary 1st stage	22 (14%)	43 (23%)	40 (24%)	37 (22%)	45 (23%)	22 (14%)	39 (23%)	82 (24%)
Level 3: Secondary second	15 (9%)	7 (4%)	20 (12%)	20 (12%)	14 (7%)	16 (10%)	14 (8%)	22 (6%)
Level 4: Post-secondary	37 (22%)	23 (13%)	18 (11%)	18 (10%)	26 (14%)	19 (12%)	28 (16%)	51 (15%)
Level 5: Tertiary	63 (38%)	46 (25%)	69 (42%)	66 (39%)	81 (42%)	69 (44%)	62 (36%)	90 (26%)
Other	2 (1%)	0	0	0	0	0	0	0
Missing	7 (4%)	18 (10%)	1 (1%)	6 (4%)	8 (4%)	8 (5%)	6 (4%)	17 (5%)
Current medication:								
Not on DMARDs	89 (54%)	n/a	56 (34%)	143 (84%)	n/a	n/a	113 (66%)	
Monotherapy	11 (7%)		76 (46%)	25 (15%)			52 (31%)	
Combination therapy	0		29 (18%)	2 (1%)			2 (1%)	
Biologic drugs	65 (39%)		3 (2%)	0			2 (1%)	
Missing	0		0	0			2 (1%)	

Key: *AS* Ankylosing spondylitis; *OA* Osteoarthritis; *SLE* Systemic Lupus Erythematosus; *SS* Systemic Sclerosis; *CP/FM* Chronic Pain/ Fibromyalgia;

*CHUL* chronic hand/upper limb conditions; *PSS* Primary Sjorgens Syndrome; *RA* Rheumatoid Arthritis; *ISCED* International Standard Classification of Education; *DMARDs* Disease Modifying Anti-Rheumatic Drugs

In general, all British English VLAs items were deemed important and relevant. In terms of comprehensibility, item 13 “going to social events, parties, or celebrations” and item 18 “taking part in leisure activities OUTSIDE your home, such as going to the pub, bingo, going to the cinema, club meetings, restaurants” raised the question whether these are measuring the same concept amongst most participants (*n* = 21) as they required similar considerations to be able to participate. For example, participants noted participation depended on location and accessibility. Several participants (*n* = 8) queried whether item 21 [driving or getting around your community by public transport] should be divided into separate items as they perceived “driving” and “using public transport” different transport options. However, when explained that this item measures participation (i.e. at a societal level) rather than activity limitation (i.e. at a personal level) they did not think it needed to change. Two participants suggested that item 27 (taking care of social communication, such as writing letters, sending emails, making phone calls or texting) could be separated into verbal and written communication. However, as this was raised by only two out of 31 participants, the original item remained unchanged.

Participants also struggled with the question “Do you have to make changes to how you do this activity because of your arthritis?” They were unclear whether to tick ‘no’ or just leave it blank if ‘unable to do’ the activity. This issue was resolved by adding further instructions to the VLAs to aid responder’s decision making. Item 33 “having intimate relations with your spouse/ partner” was perceived as too intrusive by some participants (*n* = 6). However, as the majority of the responders found this item to be relevant and appropriate, the item was retained.

Following the cognitive de-briefing interviews, no new items were added. Instead, some changes were made to the layout and wording of the items, so they are relevant and comprehensible to the British population (Additional File 2). The changes made were submitted to the lead developer who agreed to these, as these were acknowledged as differences in expression between North American and British English.

### Phase-2: psychometric testing

In Phase-2, 1929 NHS patients were screened, and a further 3365 invitations were sent through Patient Organisations (Fig. 1). From both of these sources, 1946 were interested and eligible, of whom the most (97%) consented; and 1546 (81%) returned the postal questionnaire. The participants’ socio-demographic and health characteristics are detailed in Tables 1 and 2. The response options to all 33 items are shown in Table 3, including the percentage of those reporting that an item “Does not apply to me”. Only 79 (5%) respondents completed all 33 items (including the “does not apply to me” option).

**Table 2:** The British VLAs Participants’ Health Characteristics

	AS (n = 165)	OA (n = 184)	SLE (n = 164)	SS (n = 170)	CP (n = 194)	CH (*n* = 157)	PSS (n = 171)	RA (n = 340)
HAQ20: 0–60 Median (IQR)	5.50 (1.00–14.00)	9.00 (4.00–19.50)	7.50 (2.00–21.0)	10.00 (30.00–20.00)	17.00 (8.25–28.00)	6.00 (1.00–15.00)	4 (0.25–13.00)	13.00 (4.00–23.00)
SF-36v2^a^ Physical Function norm scored	43.64 (30.75–49.88)	36.49 (28.83–46.06)	36.26 (26.92–46.06)	36.49 (26.92–45.58)	30.75 (23.09–38.40)	46.06 (38.40–51.80)	40.32 (30.75–49.88)	36.49 (26.93–46.06)
SF-36v2^a^ Bodily Pain norm scored	42.64 (34.58–50.71)	38.21 (30.55–42.64)	38.21 (30.55–46.68)	42.24 (38.21–49.90)	30.55 (26.52–38.21)	38.21 (33.27–62.00)	42.24 (38.21–51.51)	42.24 (34.18–47.48)
SF-36v2^a^ Vitality norm scored	43.69 (37.74–49.63)	43.69 (34.77–52.60)	37.74 (31.80–46.66)	40.72 (34.03–49.63)	34.77 (25.86–43.69)	46.66 (37.74–52.60)	40.72 (31.80–49.63)	43.69 (34.77–49.63)
SF-36v2^a^ Mental Health norm scored	48.25 (43.02–56.10)	48.25 (40.40–54.14)	45.64 (37.79–51.53)	48.25 (40.40–56.10)	43.02 (29.94–48.25)	48.25 (40.40–53.48)	45.64 (37.79–53.48)	50.8 (43.02–56.10)

Key: *HAQ* Health Assessment Questionnaire; ^a^SF36v2 = Short Form 36v2 Median (IQR) 0–100; lower scores = better in HAQ20. SF36v2 scored using Quality Metric Health Outcomes™ Scoring Software 4.5

**Table 3:** Responses to the British VLAS (*n* = 1545)

		Response option (%)				
	Item	Without Difficulty	Some Difficulty	Much Difficulty	Unable to Do	Not Apply
1	**Taking care of your basic needs, such as bathing, washing, getting dressed or taking care of personal hygiene**	48.0	39.0	8.4	1.5	3.1
2	Preparing meals and cooking	48.2	33.1	9.5	3.0	6.3
3	**Doing light housework, such as dusting or laundry**	51.8	30.2	8.0	2.6	7.4
4	**Doing heavier housework, such as vacuuming, changing sheets, or cleaning floors**	24.5	32.8	21.7	12.9	8.2
5	Doing other work around the house, e.g. making minor home repairs or working in the garage fixing things	15.5	15.4	7.4	16.4	45.2
6	**Gardening or outdoor property work**	16.3	29.3	13.5	15.0	26.0
7	**Shopping and doing errands**	40.7	34.1	13.7	5.7	5.9
8	**Going to appointments, such as going to the doctor or dentist, or going to have your hair cut or done**	61.2	21.7	9.0	1.0	7.2
9	**Taking care of young children in your family or doing things for them.**	20.1	16.1	6.4	2.0	55.5
10	**Taking part in activities with young children in your family**	16.8	20.0	8.4	2.7	52.4
11	**Taking care of other family members, such as your spouse or parent, or other people close to you**	30.1	19.0	6.9	4.2	39.8
12	Visiting friends or family members in their homes	58.1	23.2	6.3	2.7	9.7
13	Going to social events, parties, or celebrations	43.2	21.1	6.7	3.9	25.2
14	**Having friends and family members visit you inyourhome**	62.0	20.1	4.3	1.1	12.4
15	**Walking or getting around INSIDE your home**	63.7	27.6	5.1	0.4	3.2
16	**Walking OUTSIDE, just to get around, in the area around your home or other places you need to go on a regular basis (This does not include walking for exercise)**	53.6	28.9	11.7	2.0	3.8
17	**Taking part in leisure activities IN YOUR HOME, such as reading, watching television, listening to music**	78.2	14.5	3.0	0.2	4.1
18	Taking part in leisure activities OUTSIDE your home, such as going to the pub, bingo, going to the cinema, club meetings, restaurants	44.1	23.2	7.2	3.6	21.9
19	**Working on hobbies, crafts, or creative activities, such as music, knitting, sewing, woodworking, or painting**	25.6	24.7	8.7	7.2	33.9
20	**Taking part in physical recreational activities, such as walking for exercise, dancing, playing golf, bicycling, swimming or water aerobics**	20.9	31.7	12.9	15.0	19.5
21	**Driving or getting around your community by public transport**	53.9	26.5	6.7	2.8	10.2
22	**Travelling long distances**	52.7	23.7	10.5	3.9	9.2
23	**Taking part in religious or spiritual activities or religious services**	23.9	6.7	1.2	1.8	66.4
24	**Doing volunteer work**	16.6	7.6	1.6	5.1	69.1
25	**Working at a job for pay**	15.2	18.1	4.3	6.8	55.7
26	**Taking care of household business, e.g. pay bills or scheduling repairs**	66.8	14.2	3.9	1.0	14.1
27	**Taking care of social communication such as writing letters, sending e-mails, making phone calls or texting**	63.5	24.3	5.3	0.8	6.1
28	**Going to college or educational activities**	11.2	3.1	1.3	2.2	82.2
29	Sleeping	27.8	45.8	22.3	0.8	3.3
30	Eating and chewing	75.0	16.4	4.1	0.0	4.5
31	**Meeting new people**	65.6	8.6	3.1	0.9	21.8
32	**Having and/or taking care of a pet.**	25.8	13.9	2.5	1.6	56.1
33	Having intimate relations with your spouse/partner	27.4	20.8	7.6	6.8	37.4

^a^ Items in bold are those subsequently included in the VLAS-CAT25

#### Construct validity (confirmatory factor analysis and Mokken scaling)

A Confirmatory Factor Analysis failed to support a unidimensional scale from the 33-item VLAs (Chi Square 3552:df 464:*p* < 0.0001; RMSEA 0.066(90CI: 0.064–0.068); CFI .985; TLI 0.984); the 25-item VLAs (Chi Square 2836:df 275:p < 0.0001; RMSEA 0.078(90CI: 0.076–0.081); CFI .987; TLI 0.986; or the 14-item Short VLAs version (Chi Square 1228:df 77:p < 0.0001; RMSEA 0.099 (90CI: 0.094–0.104). Based on the item classification into the three domains (Obligatory, Committed, Discretionary), the three-domain structure of the item set also failed (Chi Square 2693:df 272: p < 0.0001; RMSEA 0.076(90CI: 0.074–0.079);

CFI .987; TLI 0.986). Modification indices throughout these analyses indicated substantial cross loading, particularly between Obligatory and Committed items, and substantial local dependency among pairs of items, thus requiring correlated errors. Given the ancillary fit statistics were more supportive, the results suggest that the disturbance of structure may be strongly influenced by clusters of locally dependent items. A Loevinger Coefficient from Mokken scaling of 0.87 for all 33 items indicated a strong non-parametric association between items, and despite the lack of evidence of unidimensionality (which is an assumption of Mokken), provided sufficient evidence to move forward to a Rasch analysis of the data.

#### Rasch: diagnostics

Fit of the data from the VLAs to the Rasch model is shown in Table 4. An initial Likelihood Ratio test to determine if a Rating scale or Partial Credit parameterisation was appropriate supported the latter (Chi-Square 1281.3 (df 63); p = < 0.0001). For each of the eight conditions, fit is shown for the 33, 25 and 14-item versions. Only four analyses satisfied the stochastic ordering (fit) and unidimensionality assumptions (AS-25; AS-14; SS-25; PS-14). Even here, the local independence assumption was breached by clusters of residual item correlations, although of insufficient magnitude to affect the fit and unidimensionality tests. Elsewhere, the VLAs was characterised by multidimensionality and misfit, which again may have been influenced by extensive clusters of residual item correlations. While reliability was high in all cases, this could be expected to be inflated in the presence of local response dependency, as identified through the residual correlation patterns. Differential item function was occasionally present for age, gender and marital status, but not for education or duration of condition. For example, “Doing heavy housework’ was more difficult for females at any level of ability. DIF was also present for condition in 15 of the 33 items. For example, for those with RA, ‘traveling long distances’ was more difficult than other conditions at all levels of life activity. Likewise, ‘Taking care of social communication’ was more difficult for those with chronic hand/upper limb conditions, at any level of life activity. Overall, the easiest activities (difficulty rarely affirmed) were ‘eating’ and ‘taking part in leisure activities in the home’, while the hardest activities (difficulty common) were ‘minor home repairs’ and ‘gardening’.

**Table 4:** Rasch Analysis of Various Versions of the Scale by Condition

	Condition and number of items	*Chi- Square*^a^	Df	P	Residual Item		Residual Person		PSI Reliability	% tests > 5%	95%CI	N
					Mean	SD	Mean	SD				
1	Ankylosing Spondylitis- 33	129.3	66	< 0.001	−0.5876	1.6027	−0.3703	1.0239	0.93	8.02	4.7–11.4	165
2	AS-25	54.0	50	0.324	− 0.4318	1.1425	− 0.3715	1.1252	0.92	7.64	4.2–11.1	165
3	AS-14	24.0	28	0.684	−0.3933	1.3061	− 0.4036	1.0915	0.90	5.73	2.3–9.1	165
4	Rheumatoid Arthritis-33	315.3	165	< 0.001	−0.3128	2.0134	−0.2709	1.0995	0.95	6.67	4.3–9.5	336
5	RA-25	214.2	200	0.233	−0.2082	1.5988	−0.3244	1.1197	0.95	10.64	8.3–13.0	336
6	RA-14	141.5	112	0.031	−0.2118	1.7771	−0.4067	1.1493	0.92	8.51	6.2–10.9	336
7	Chronic Pain −33	210.49	132	< 0.001	−0.2716	1.6226	−0.2152	1.2471	0.96	8.65	5.5–11.8	194
8	CP-25	121.7	100	0.069	−0.2432	1.3791	−0.2654	1.2142	0.96	9.73	6.6–12.9	194
9	CP-14	77.1	56	0.032	−0.1707	1.3792	−0.2721	1.1337	0.93	10.38	7.2–13.5	194
10	Chronic Hand/Upper Limb-33	145.9	66	< 0.001	−0.2638	1.7283	−0.2785	0.9902	0.91	7.95	4.5–11.4	157
11	CH-25	76.0	50	0.010	−0.1936	1.4952	− 0.3153	1.0373	0.91	8.61	5.1–12.1	157
12	CH-14	44.4	28	0.026	−0.1760	1.5444	−0.2875	0.8676	0.88	9.15	5.7–12.6	157
13	Osteoarthritis-33	156.4	99	< 0.001	−0.3829	1.4713	−0.2158	0.8122	0.94	10.92	7.7–14.2	184
14	OA-25	94.5	75	0.063	−0.3394	1.1947	−0.2598	0.8926	0.94	12..07	8.8–15.3	184
15	OA-14	52.3	42	0.133	−0.3406	1.1186	−0.2979	0.9932	0.91	12.07	8.8–15.3	184
16	Systemic Lupus −33	201.5	99	< 0.001	− 0.2439	1.7943	−0.1998	1.1412	0.94	6.17	2.8–9.5	164
17	SLE-25	148.7	75	< 0.001	−0.3171	1.4556	− 0.2224	1.0881	0.94	9.92	6.5–13.2	164
18	SLE-14	67.5	42	0.007	−0.2797	1.3644	−0.3106	1.1546	0.91	10.43	7.1–13.8	164
19	Systemic Sclerosis-33	214.7	99	< 0.001	−0.2154	1.7710	−0.2079	1.0446	0.91	6.21	2.7–9.8	170
20	SS-25	88.4	75	0.139	−0.1269	1.4387	−0.2573	1.0479	0.91	6.45	3.0–9.9	170
21	SS-14	45.7	42	0.321	−0.1567	1.2816	−0.3223	1.0401	0.87	8.43	5.1–11.7	170
22	Primary Sjogren’s-33	255.8	99	< 0.001	−0.3904	1.7979	−0.2317	1.1495	0.90	7.88	4.5–11.2	171
23	PS-25	138.8	75	< 0.001	−0.2100	1.2663	−0.3023	1.1632	0.90	4.85	1.5–8.2	171
24	PS-14	50.5	42	0.173	−0.1771	1.1396	−0.4102	1.1496	0.87	6.21	2.8–9.6	171
	**Ideal Values**			**> 0.05**^a^		**< 1.4**		**< 1.4**	**> 0.70**	**< 5.0**	**LCI < 5.0**	

^a^Bonferroni Adjusted (for 33 & 25 items fit is 0.002; for 14 items is 0.004)

The clusters of locally dependent items did not necessarily conform to the Obligatory, Committed or Discretionary domains. For example, in people with RA, the items ‘doing other work around the house’ and ‘gardening or outdoor property work’ were designated as Committed and Discretionary respectfully, displayed a residual correlation of 0.506 in the 33-item version, and 0.447 in the 25-item version. Nevertheless, fit to the model constrained to within the domains showed some improvement, although the occasional misfit and multidimensionality remained (Table 5). This suggests that much of the disturbance of fit and dimensionality could be attributable to the local dependency issue.

**Table 5:** Rasch Analysis of the Domain Scores

	Condition and number of items	Chi- Square*	Df	P	Residual Item		Residual Person		PSI Reliability	% tests > 5%	95% CI	N of cases/items
					Mean	SD	Mean	SD				
	**Rheumatoid Arthritis**											**336**
1	Obligatory	31.6	25	0.171	−0.0025	0.7371	−0.3163	0.8669	0.79	1.84	0.0–4.2	5
2.	Committed	41.3	40	0.415	−0.3659	0.8992	− 0.5156	1.2163	0.88	5.0	2.5–7.3	8
3.	Discretionary	59.6	55	0.313	−0.3283	1.1107	−0.3196	0.8450	0.86	4.8	2.4–7.2	11
4	Activities	74.2	65	0.203	−0.3421	1.0915	−0.4057	1.1325	0.91	7.32	5.0–9.7	13
	**Ankylosing Spondylitis**											**165**
5	Obligatory	32.5	35	0.590	−0.4068	0.9407	−0.4738	1.0066	0.70	3.75	1.0–8.5	5
6	Committed	22.4	24	0.558	−0.0942	0.9214	−0.3141	1.0133	0.86	5.63	2.2–9.0	8
7	Discretionary	43.2	33	0.110	−0.3089	1.3239	−0.3631	0.8425	0.82	5.63	2.2–9.0	11
8	Activities	98.7	117	0.888	−0.2960	0.8862	−0.3474	1.0455	0.89	4.94	1.6–8.3	13
	**Chronic Pain/FM**											**194**
9	Obligatory	13.7	20	0.846	−0.0021	0.6141	−0.4108	1.1276	0.86	4.49	1.1–7.9	5
10	Committed	19.8	24	0.708	−0.2349	1.0388	−0.4173	1.1389	0.90	5.35	2.2–8.5	8
11	Discretionary	45.0	33	0.079	−0.4593	1.6245	−0.3326	0.9164	0.87	4.32	1.2–7.5	11
12	Activities	62.2	39	0.010	−0.2450	0.9657	−0.2855	1.1148	0.94	8.95	5.8–12.0	13
	**Chronic HUL**											**157**
13	Obligatory	18.7	15	0.229	−0.3316	1.2499	− 0.3862	0.7203	0.50	2.65	0.0–6.1	5
14	Committed	26.0	24	0.355	−0.3128	1.5619	−0.4211	0.8959	0.88	7.52	3.8–11.2	8
15	Discretionary	32.8	33	0.475	−0.3164	0.9507	−0.4290	1.0548	0.76	3.42	0.0–6.6	11
16	Activities	50.2	39	0.108	−0.1920	1.5876	−0.3047	0.8493	0.88	7.24	3.8–10.7	13
	**Osteoarthritis**											**184**
17	Obligatory	31.1	15	0.009	−0.0317	1.2638	−0.2855	0.8517	0.78	2.85	0.0–6.1	5
18	Committed	27.6	24	0.270	−0.3113	0.6352	−0.3043	0.8110	0.87	5.26	1.8–8.7	8
19	Discretionary	49.7	33	0.031	−0.4230	1.2061	−0.3355	0.8260	0.83	5.49	2.2–8.8	11
20	Activities	31.3	39	0.807	−0.3510	0.8760	−0.2667	0.8752	0.91	10.92	7.7–14.2	13
	**Systemic Lupus**											**164**
21	Obligatory	15.2	15	0.440	−0.0414	0.5008	−0.3181	0.9331	0.77	3.07	0.0–6.4	5
22	Committed	22.4	24	0.558	−0.3746	0.9971	−0.4169	1.002	0.90	4.91	1.6–8.3	8
23	Discretionary	40.0	33	0.036	−0.3000	1.2608	−0.2669	0.8150	0.84	5.56	2.2–8.9	11
24	Activities	50.1	39	0.110	−0.5035	1.0784	−0.2664	0.9472	0.92	8.59	5.2–11.9	13
	**Systemic Sclerosis**											**170**
25	Obligatory	23.4	15	0.075	−0.0035	1.4896	−0.2991	0.8805	0.62	3.00	0.0–6.3	5
26	Committed	25.2	24	0.395	−0.0556	0.8197	−0.3076	0.8682	0.86	3.03	0.0–6.4	8
27	Discretionary	46.2	33	0.063	−0.1914	1.4057	−0.2351	0.7523	0.80	2.45	−0.09-5.8	11
28	Activities	41.6	39	0.358	−0.1652	1.1150	−0.3109	0.9900	0.87	4.85	1.5–8.2	13
	**Primary Sjogren’s**											**171**
29	Obligatory	12.2	10	0.273	0.0650	1.2408	−0.1897	0.9149	0.55	1.81	−1.5-5.1	5
30	Committed	31.8	24	0.131	−0.1624	0.9641	−0.4212	0.8971	0.85	2.45	−0.9-5.8	8
31	Discretionary	4.1	33	0.094	−0.1784	1.2859	−0.3196	0.8102	0.74	0.61	−2.7-3.9	11
32	Activities	46.6	39	0.189	−0.4608	1.1457	−0.4803	1.0434	0.86	3.03	0.0–6.4	13
	**Ideal Values**			**> 0.05**		**< 1.4**		**< 1.4**	**> 0.70**	**< 5.0**	**LCI < 5.0**	

#### An item Bank approach

**Fig. 2:**
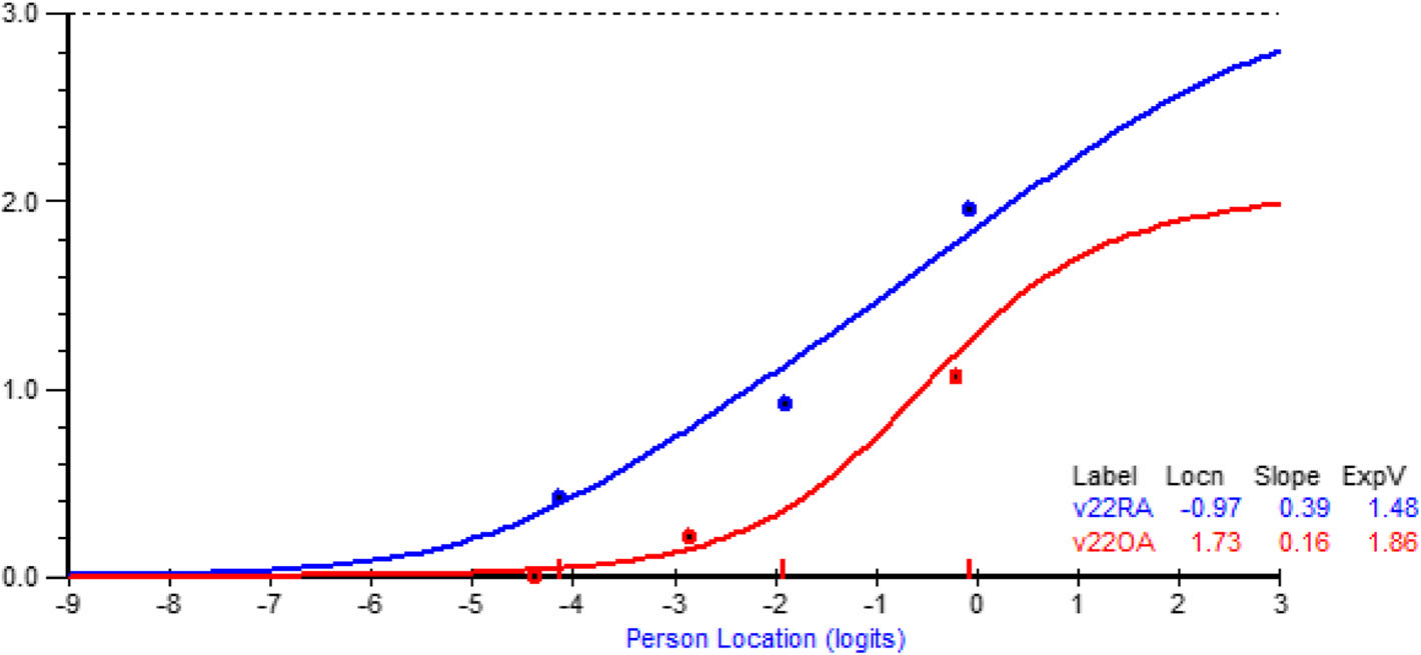
Comparison of RA & OA on response to traveling long distances

**Fig. 3:**
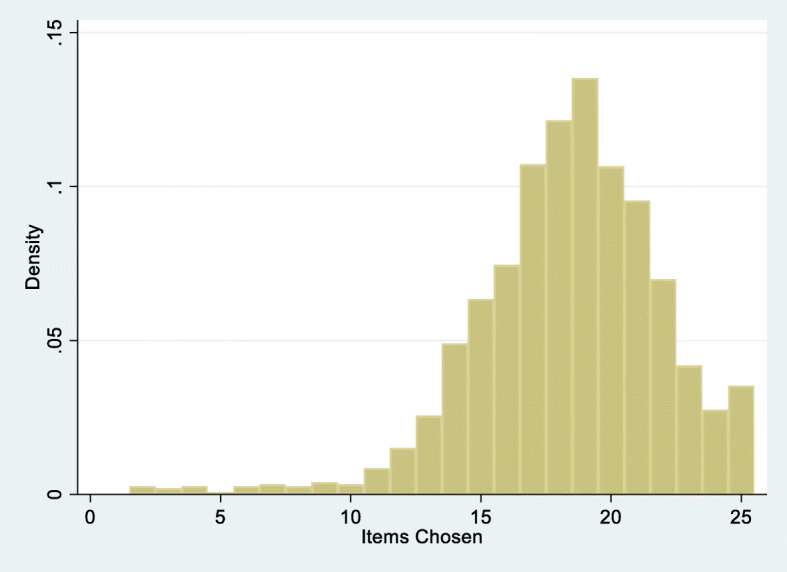
Distribution of items chosen from the VLAs-25 CAT item set

Consequently, the item bank approach was applied. A core set of 15 items were shown to fit the Rasch model across most indicators (Table 6, Analyses 1–3). However, the item ‘Travelling long distances’ showed DIF by condition with, for example, RA and OA showing distinct differences in expected response at any level of difficulty, the former having more difficulty than the latter (Fig. 2). Having set aside the surplus items from the local dependency analysis, a second item set was created with 10 items, which again showed fit to the model and no DIF by condition (Table 6. Analyses 4–6). Thus, 25 of the 33 items were available for CAT, and with the second set calibration anchored by three items from the core set, all items were calibrated onto the same unidimensional interval scale metric. The mean number of items chosen (i.e. excluding “does not apply to me” responses) from the VLA-CAT25 in the main data set was 17.4, and the maximum was 24 (3.5%) (Fig. 3). Simulation of the efficacy of the CAT identified that for group use the average number of items required to achieve an alpha of ≥0.7 was 4, and 11 to achieve an alpha of 0.85 for individual use. Consequently, it would appear that given the patient choice of relevant items, the CAT can in most cases accommodate both individual and group estimates with the required reliability, should the distribution shown in Fig. 3 be replicated elsewhere.

**Table 6:** Rasch Analysis of Computer Adaptive Testing item sets

Analysis	Item Sets and samples	***Chi- Square***^a^	Df	P	Residual Item	Residual Person	PSI Reliability	% tests >5%	95%LCI	N
					SD	SD				
1	Core Set- CAT15 Sample 1	153.0	135	0.138	1.586	0.967	0.87	6.8	4.7	500
2	Core Set- CAT15 Sample 2	172.5	135	0.016	1.711	0.934	0.88	6.6	4.5	500
3	Core Set- CAT15 Sample 3	165.2	135	0.040	1.576	0.880	0.88	5.1	3.0	500
4	Second Set- CAT10 Sample 1	106.6	90	0.112	1.314	0.927	0.84	5.6	3.2	500
5	Second Set- CAT10 Sample 2	114.2	90	0.043	1.694	0.993	0.85	3.6	1.1	500
6	Second Set- CAT10 Sample 3	106.6	90	0.112	1.498	1.021	0.86	5.2	3.0	500
	**Ideal Values**		**>0.05**^a^		**<1.4**	**<1.4**	**>0.70**	**<5.0**	**<5.0**	

^a^Bonferroni Adjusted (0.008 for 6 tests)

### Summary of the results

The 33-item VLAs was linguistically validated and culturally adapted for British people aged ≥18 years with RMDs following recommended guidelines. The British English VLAs retained all of the original 33-items, with some changes to the wording, template and instructions to make it easily understandable by British people. Following this, the VLAs was tested in its 33, 26 and 14 item versions with British people across 8 different RMDs to verify its psychometric validity and reliability (internal consistency). The latter two versions were nested within the 33-item version, with a minor change to the 26-item version which had split an original item into two parts. The results of the statistical analysis show that the VLAs, in its various summated forms (i.e. adding together items in complete sets scored using those items identified as important to the person) was not a valid measure of valued life activities. Only 5% of the sample considered all the items applied to them.

When a calibration was made for use in a CAT, 25 of the 33 items were retained, and formed a valid unidimensional item set, largely invariant by condition. The CAT could provide sufficient reliability to accommodate both individual and group estimates. Using suitable CAT software, these items could be administered taking account of both the varying difficulty of the items, the local dependency that exists, and the DIF on the ‘travel’ item, so giving an estimate of VLA on an 0–100 interval scale, irrespective of the number of items chosen.

## Discussion

The Valued Life Activities scale was completed by a large number of people across eight RMDs. The VLAs was perceived as a relevant and understandable measure of activities and participation by British people with RMDs. However, robust psychometric testing of the British VLAs in the context of the current scoring method (i.e. summing items identified as important to the respondent only) of the 33, 25 and 14 item versions showed that, due to local item dependency, multidimensionality and misfit to Rasch model expectations, the VLAs had insufficient validity to enable a recommendation for its use as summated item sets in clinical evaluation or research. The usual strategy of scoring only those items that apply to the individual does not exempt the underlying item set from basic psychometric requirements, as the choices that are made deliver an almost infinite subset of items from the whole, each of which should satisfy those same requirements.

The ‘Does not apply to me’ response also raises substantial problems with how these items are scored, and how to deal with this response (in addition to any other type of missingness). The problem is similar to that observed for Goal Attainment Scaling where patients are involved with the choice of goals for their rehabilitation [42]. While Rasch analysis can deal with both structural and ordinary missingness, and multiple imputation techniques can provide complete data sets, this is unlikely to be available in routine clinical practice [43]. Also, imputation techniques are not designed to deal with ‘missing not at random’ instances, which is likely to be the case with the ‘does not apply to me’ option. Furthermore, the usual strategy for scales to provide a transformation table from raw score to interval scaled Rasch metric would also not apply, as it is only valid in the presence of complete data, which is not attainable under the present scoring method. This also affected actions to remedy the effects of local dependency, that is by creating ‘super items’ (testlets) by adding together clusters of items, as the ‘Does not apply to me’ option resulted in case-wise deletion at the testlet level. Given these problems, it was not possible to test for DIF cancellation at the scale level due to the restriction upon creating testlets [44]. Furthermore, under the current scoring method, DIF would have to be assessed across all possible combinations of items to examine if any DIF is observed, and would cancel across the chosen items, given the person estimate would be re-estimated for each unique combination of items.

Some of the above problems were accommodated through a CAT design, identifying 25 items (in two sets of 15 and 10) which demonstrated fit to the Rasch model, including unidimensionality and invariance by most contextual groups. The DIF by condition for the ‘travel’ item needed a condition-specific item location estimate for those conditions affected. The calibrated item set, given suitable CAT software, could be administered to patients, offering the option of ‘not important for me’ and ‘not applicable to me’.

### Implications for clinical and research practice

The main implication for clinical and research practice is that the implementation of the above solution requires access to CAT software or some system to provide CAT-based estimates, and appropriate IT infrastructure at the clinic level, or at least that the patient has online facilities at home. One application, the smartCAT system, was designed to facilitate such an environment, but requires on-line interaction with its server, which will return an estimate in real time to the source, including an appropriate clinical setting, as required [45]. It can cope with clusters of locally dependent items, and different estimates to account for DIF where present. Another CAT solution can be found with the Concerto software, which is an open-source online adapting testing platform https://concertoplatform.com/about [46]. The former has a small charge per assessment, while the latter is free, but psychometric and technical applications can be supported as required for a fee. So, when suitable software is available, using the VLAS in this manner addresses the EULAR recommendations of assessing patient’s preferences and priorities concerning the impact upon their activities and participation.

### Limitations

We only conducted cognitive debriefing interviews with people with RA, predominantly from the North West and Midlands regions of England, due to budget and timeline constraints. However, we tested the psychometric properties of the VLAs amongst eight RMDs. Cognitive debriefing with people with other RMDs may have resulted in reduction or addition of new items to the British VLAs.

We intended to also examine test-retest reliability. After 4 weeks of completing the first questionnaire booklet, participants were mailed a second including the VLAs. However, as the Rasch analysis identified significant challenges in calculating scores, we did not progress to test this. There is no reason why a ‘stable’ respondent should choose the same set of items, even within a short time frame. Given the potential number of item sets that could be chosen, the retest can only be done on those who have completed exactly the same set of items across time. The question then arises as to whether or not a failure to choose the same set of items constitutes a lack of test-retest reliability. Even where the same set of items are chosen, given the possible number of combinations available, each combination should have sufficient cases for the analysis, as though they were distinct scales. Further work is required to consider how test-retest reliability may be undertaken in such circumstances.

The VLA-CAT25 item bank itself has only just been developed within this study and will require further psychometric testing and testing in clinical settings to ascertain how well it works in a day-to-day setting. The smartCAT software is at its Beta test stage but has been trialled on a fatigue item bank in a clinical setting in Sweden. It requires careful management of the CAT process, assigning unique patient identifiers, setting up CAT in clinic, or providing links and passwords for use at home. Data protection must be considered as the estimate itself is created on the smartCAT server located outside of the European Union and delivered in real time back to the source or designated setting. Decisions need to be taken as to whether or not the estimate and its associated patient identifier is stored on the foreign server, or not. Similar software programmes are likely to have the same requirements.

The simulation of fit to the Rasch model was not ideal. For example, the distribution of 100 random samples would give more accurate picture of fit of the item bank item sets, rather than just three consecutive samples with replacement. Unfortunately, this option is not available in the software used. Consequently, further work would need to verify the 15 & 10 items set fit. It is not possible to test the fit of the 25 items together, as the second set holds items which were locally dependent with the core set, and which would generate multidimensionality and misfit.

## Conclusions

The British version of the VLAs, across various scales, failed to satisfy classical and modern psychometric standards as full item sets. A CAT solution was found that would overcome these limitations and avoid using inappropriate mathematical operations to derive a VLAs score. It provides a Rasch derived interval scaled estimate of Valued Life Activities from the set of items chosen by the participant. Increasingly available CAT software is required to implement the item bank. Further validation of the CAT application is required.

## Supplementary information

**Additional file 1.** VLAs Published Versions and Psychometric Testing.

**Additional file 2.** Original VLAs items vs the British VLAs items.

**Additional file 3.** British Valued Life Activities Scale [British VLAs].

## Abbreviations

AS: Ankylosing Spondylitis
CAT: Computer Adaptive Testing
CP: Chronic Pain
CUL: Chronic Upper Limb Pain
CFI: Comparative Fit Index
CFA: Confirmatory Factor Analysis
DIF: Differential Item Functioning
DMARDs: Disease Modifying Anti-Rheumatic Drugs
EULAR: European League Against Rheumatism
FM: Fibromyalgia
HAQ: Health Assessment Questionnaire
NHS: National Health Service
NRS: Numeric Rating Scale
PROMs: Patient Reported Outcome Measures
PSI: Person Separation Index
PSS: Primary Sjogren’s Syndrome
RA: Rheumatoid Arthritis
OA: Osteoarthritis
SF-36_v2_: Short-Form 36
SD: Standard Deviation
SLE: Systemic Lupus Erythematosus
SS: Systemic Sclerosis
TLI: Tucker Lewis Index
UK: United Kingdom
