# Supplementary material for: The Valued Life Activities Scale (VLAs): linguistic validation, cultural adaptation and psychometric testing in people with rheumatic and musculoskeletal diseases in the UK
Source: BMC Musculoskelet Disord. 2020 Jul 30;21:505. doi: 10.1186/s12891-020-03409-9 (PMC7393896; doi:10.1186/s12891-020-03409-9)
Supplement: Supplementary file 1 — Additional file 1. VLAs Published Versions and Psychometric Testing. [file 12891_2020_3409_MOESM1_ESM.docx]

**Additional File 1. VLAs Published Versions and Psychometric Testing**

|  | **Original VLAs 33** | **Jette and Verbrugge domain**  ***Sub-scale:**  Committed [C]  Discretionary [D]  Obligatory [O] | **VLAs 29**  **+** item 23 is divided into two separate items. | **VLAs 26**  **+** item 23 is divided into two separate items. | **VLAs 21**  **+** item 23 is divided into two separate items. | **VLAs 14**  **(Short VLAs)** | **Psychometric Testing** |
| --- | --- | --- | --- | --- | --- | --- | --- |
| **1** | Basic needs | Obligatory | √ | √ | √ | √ | *In all versions activities that are not applicable/ not important are not scored.  Scoring:  5-point scale 0-4 (no difficulty to unable to perform)  Other ratings increased by 1 to account for the additional category  Total score= mean of items difficulties.  **1) Preliminary Validation for The S-Vla Eular 2010** (Katz Ann Rheum Dis 2005;64(Suppl III):402)  (27 activities) n=84???  HAQ scores were significantly (p<.0001) associated with both VLA scores in bivariate models (# VLAs affected, R2=.25, beta[b]=5.0; mean difficulty, R2=.64, b=3.2), as were PF scores (# VLAs affected, R2=.11, b=-.08, p=.003; mean difficulty, R2=.39, b=-0.06). DAS28 was also significantly associated with VLA difficulty (b=.48, R2=.05, p=.04), but not with number of VLAs affected (p=.85). When DAS28 was added to regression models of mean VLA difficulty to test mediation, both HAQ and PF remained significant in individual models, but in both cases, DAS28 was no longer significant.  **2) Preliminary Validation For The S-Vla Acr 2009** (Katz et al Arthritis Rheum 2009;60: S421)  (n=100)  *Cronbach’s alpha= .0.93  *All items were strongly correlated with the S-VLA total score (ranging from 0.59-0.79). Total S-VLA was highly positively correlated with patient-reported disease activity (RADAI [r=0.71; p<=0.001], the HAQ-D [r=0.77; p<=0.001], SAWS total score [r=0.77; p<=0.001], SAWS satisfaction with abilities subscale [r=0.77; p<=0.001] and APaQ days with activity limitations [r=0.61; p<=0.001]). the S-VLA was significantly inversely correlated with SF-36 Physical Component Summary score (r=-0.76; p<=0.001) and the SF-36 subscales: Physical Functioning (r=–0.75; p<=0.001), Role Physical (r=–0.68; p<=0.001) and Social Functioning (r=–0.71; p<=0.001). |
| **2** | Meals/cook | Committed | √ | √ | √ | √ |  |
| **3** | Light housework | Committed | √ | √ |  | √ |  |
| **4** | Heavy housework | Committed | √ | √ | √ | √ |  |
| **5** | Minor repairs | Committed | √ | √ | √ |  |  |
| **6** | Gardening/ property work | Discretionary | √ | √ |  | √ |  |
| **7** | Administration/Household b. | Committed | √ |  |  |  |  |
| **8** | Walking inside | Obligatory | √ | √ |  | √ |  |
| **9** | Walking outside | Obligatory | √ | √ | √ | √ |  |
| **10** | Getting around your comm. | Obligatory | √ | √ | √ |  |  |
| **11** | Going to appointments | Committed | √ | √ |  |  |  |
| **12** | Shopping | Committed | √ | √ | √ |  |  |
| **13** | Childcare | Committed | √ | √ |  |  |  |
| **14** | Activities with children | Discretionary | √ | √ | √ |  |  |
| **15** | Other family care | Committed | √ | √ | √ | √ |  |
| **16** | Social events | Discretionary | √ | √ | √ | √ |  |
| **17** | Social communication | Discretionary | √ |  |  |  |  |
| **18** | Visit others | Discretionary | √ | √ | √ |  |  |
| **19** | Having others visit | Discretionary | √ | √ | √ |  |  |
| **20** | Hobbies | Discretionary | √ | √ | √ | √ |  |
| **21** | Leisure in home | Discretionary | √ | √ |  |  |  |
| **22** | Leisure activities out of home | Discretionary | √ | √ | √ | √ |  |
| **23** | Physical activities | Discretionary | √ | √ | √ | √ |  |
| **24** | Travel | Discretionary | √ | √ | √ | √ |  |
| **25** | Religious /spiritual activities | Discretionary | √ | √ | √ |  |  |
| **26** | Volunteer work | Discretionary | √ | √ |  |  |  |
| **27** | Study | Discretionary | √ |  |  |  |  |
| **28** | Working | Committed | √ | √ | √ | √ |  |
| **29** | Sleeping | Obligatory |  |  | √ |  |  |
| **30** | Eating | Obligatory |  |  |  |  |  |
| **31** | Intimate relations | Discretionary |  |  | √ |  |  |
| **32** | Meet new people | Discretionary |  |  |  |  |  |
| **33** | Care for pets | Discretionary |  |  |  |  |  |
| **3) Preliminary Validation for the S-Vla Eular 2010** (Hassett et al Ann Rheum Dis 2010;69(Suppl3):478)  (n=49)  *Cronbach’s alpha= .0.91  *Convergent and discriminant validity were evaluated using correlations between S-VLA and other measurements below.  *Strong correlations with RADAI, HAQ, SF-36, SAWS and APQ (ranging from 0.50-0.78) with the exception of one item (<0.50 (item 6: r=0.49). The S-VLA was inversely correlated with SF36 PCS and PF, RP and SF.  **4) Development and Validation of a Short Form of the Valued Life Activities Disability Questionnaire for Rheumatoid Arthritis** (Arthritis Care Res (Hoboken). 2011 December ; 63(12): 1664–1671. doi:10.1002/acr.20617)  Objective—Develop and validate a shortened version of the Valued Life Activities disability and accommodations scale (VLA) for individuals with rheumatoid arthritis (RA).  Methods—To shorten the existing VLA measure, item response theory analyses were conducted using data from 449 patients with RA. Next, the resulting 14-item shortened version of the VLA scale (S-VLA) was evaluated by structured interviews among 20 RA patients. Lastly, the S-VLA was administered to 150 RA patients along with other  measures including the Health Assessment Questionnaire (HAQ) and SF-36. A random sample of 50 patients completed the S-VLA two weeks later to assess reliability. Item statistics were calculated to evaluate correlations between individual items and S-VLA total score. Correlations between the S-VLA and other measures were used to evaluate validity.  Results—Test–retest reliability was 0.91, while Cronbach’s alpha for the S-VLA was 0.95. None of the 14 items were associated with improved alpha coefficients when omitted. All items were strongly correlated with the S-VLA total score. S-VLA scores were highly positively correlated with HAQ (r=0.81; p≤0.001), patient-reported disease activity (r=0.71; p≤0.001), satisfaction with abilities (r=0.82; p≤0.001), and number of days with activity limitations (r=0.65; p≤0.001). In addition, as hypothesized, the S-VLA was inversely correlated with SF-36 Physical Component  Summary score (r=−0.78; p≤0.001) and subscales: Physical Functioning (r=−0.80; p≤0.001), Role Physical (r=−0.67; p≤0.001) and Social Functioning (r=−0.72; p≤0.001). | | | | | | | |

***Sub-scale=** C, committed; D, discretionary, O, obligatory [Sub-scales were only shown for the 29-item VLAs by Katz, Morris and Yelin, Arthritis and Rheumatism 2008: 59 (10):1416–1423
