# Supplementary material for: The Valued Life Activities Scale (VLAs): linguistic validation, cultural adaptation and psychometric testing in people with rheumatic and musculoskeletal diseases in the UK
Source: BMC Musculoskelet Disord. 2020 Jul 30;21:505. doi: 10.1186/s12891-020-03409-9 (PMC7393896; doi:10.1186/s12891-020-03409-9)
Supplement: Supplementary file 2 — Additional file 2. Original VLAs items vs the British VLAs items. [file 12891_2020_3409_MOESM2_ESM.docx]

**Additional File 2. Original VLAs items vs the British VLAs items**

|  | **Original VLAs - 33** |  | **British VLAs -33** |  |
| --- | --- | --- | --- | --- |
| **1** | Basic needs | √ | Basic needs | **1** |
| **2** | Meals/cook | √ | Meals/cook | **2** |
| **3** | Light housework | √ | Light housework | **3** |
| **4** | Heavy housework | √ | Heavy housework | **4** |
| **5** | Minor repairs | √ | Minor home repairs | **5** |
| **6** | Gardening/ property work | √ | Gardening/ property work | **6** |
| **7** | Administration/Household b. | √ | Shopping | **7** |
| **8** | Walking inside | √ | Going to appointments | **8** |
| **9** | Walking outside | √ | Taking care of family | **9** |
| **10** | Getting around your comm. | √ | Activities with children | **10** |
| **11** | Going to appointments | √ | Taking care of other family | **11** |
| **12** | Shopping | √ | Visiting friends or family | **12** |
| **13** | Childcare | √ | Going to social events, parties | **13** |
| **14** | Activities with children | √ | Having friends/ family visiting | **14** |
| **15** | Other family care | √ | Walking inside your home | **15** |
| **16** | Social events | √ | Walking outside | **16** |
| **17** | Social communication | √ | Leisure activities at home | **17** |
| **18** | Visit others | √ | Leisure activities out of home | **18** |
| **19** | Having others visit | √ | Hobbies | **19** |
| **20** | Hobbies | √ | Physical recreational activities | **20** |
| **21** | Leisure in home | √ | Driving/ using public transport | **21** |
| **22** | Leisure activities out of home | √ | Travelling long distances | **22** |
| **23** | Physical activities | √ | Religious /spiritual activities | **23** |
| **24** | Travel | √ | Doing volunteer work | **24** |
| **25** | Religious /spiritual activities | √ | Working at a job for pay | **25** |
| **26** | Volunteer work | √ | Household business | **26** |
| **27** | Study | √ | Social communication | **27** |
| **28** | Working | √ | Educational activities | **28** |
| **29** | Sleeping | √ | Sleeping | **29** |
| **30** | Eating | √ | Eating | **30** |
| **31** | Intimate relations | √ | Meet new people | **31** |
| **32** | Meet new people | √ | Care for pets | **32** |
| **33** | Care for pets | √ | Intimate relations | **33** |
