# Supplementary material for: The Valued Life Activities Scale (VLAs): linguistic validation, cultural adaptation and psychometric testing in people with rheumatic and musculoskeletal diseases in the UK
Source: BMC Musculoskelet Disord. 2020 Jul 30;21:505. doi: 10.1186/s12891-020-03409-9 (PMC7393896; doi:10.1186/s12891-020-03409-9)
Supplement: Supplementary file 3 — Additional file 3. British Valued Life Activities Scale [British VLAs]. [file 12891_2020_3409_MOESM3_ESM.docx]

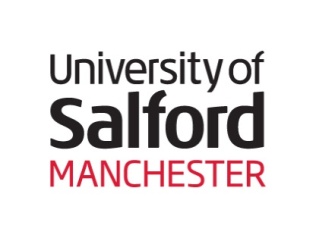


British Valued Life Activities Scale [British VLAs]

These questions are about how your arthritis affects your ability to do things that are **important** to you.

If you do not do an activity for reasons other than your arthritis, you should mark “Does not apply to me” and go to the next activity

Please indicate how much difficulty you have had over the past week with each of these activities because of your arthritis.

If an activity is not important to you, you should mark “Not important to me.”

In the last column, tick if you have to make any changes as to how you do the activity because of your arthritis. For example, do you have to:

- Limit the time you spend in this activity or how often you do it?
   - Do the activity more slowly or take more time? 
   - Get help from another person?
   - Make other changes to how you perform the activity, such as using equipment or gadgets?

Because of your arthritis how much difficulty have you had with each of the following activities over the past week?

|  | **Does not apply to me** | **No difficulty** | **Some difficulty** | **Much difficulty** | **Unable to do** | **Not important  to me** | **Do you have to make**  **changes to how you do this activity because of your arthritis?**  **Yes No** |
| --- | --- | --- | --- | --- | --- | --- | --- |
| 1. Taking care of your basic needs, such as bathing, washing, getting dressed or taking care of personal hygiene | 🞏 | 🞏 | 🞏 | 🞏 | 🞏 | 🞏 | 🞏 🞏 |
| 1. Preparing meals and cooking | 🞏 | 🞏 | 🞏 | 🞏 | 🞏 | 🞏 | 🞏 🞏 |
| 1. Doing light housework, such as dusting or laundry | 🞏 | 🞏 | 🞏 | 🞏 | 🞏 | 🞏 | 🞏 🞏 |
| 1. Doing heavier housework, such as vacuuming, changing sheets, or cleaning floors | 🞏 | 🞏 | 🞏 | 🞏 | 🞏 | 🞏 | 🞏 🞏 |
| 1. Doing other work around the house, e.g. making minor home repairs or working in the garage fixing things | 🞏 | 🞏 | 🞏 | 🞏 | 🞏 | 🞏 | 🞏 🞏 |
| 1. Gardening or outdoor property work | 🞏 | 🞏 | 🞏 | 🞏 | 🞏 | 🞏 | 🞏 🞏 |
| 1. Shopping and doing errands | 🞏 | 🞏 | 🞏 | 🞏 | 🞏 | 🞏 | 🞏 🞏 |
| 1. Going to appointments, such as going to the doctor or dentist, or going to have your hair cut or done | 🞏 | 🞏 | 🞏 | 🞏 | 🞏 | 🞏 | 🞏 🞏 |
| 1. Taking care of young children in your family or doing things for them. | 🞏 | 🞏 | 🞏 | 🞏 | 🞏 | 🞏 | 🞏 🞏 |
| 1. Taking part in activities with young children in your family | 🞏 | 🞏 | 🞏 | 🞏 | 🞏 | 🞏 | 🞏 🞏 |
| 1. Taking care of other family members, such as your spouse or parent, or other people close to you | 🞏 | 🞏 | 🞏 | 🞏 | 🞏 | 🞏 | 🞏 🞏 |

Because of your arthritis how much difficulty have you had with each of the following activities over the past week?

|  | **Does not apply to me** | **No difficulty** | **Some difficulty** | **Much difficulty** | **Unable to do** | **Not important  to me** | **Do you have to make**  **changes to how you do this activity because of your arthritis?**  **Yes No** |
| --- | --- | --- | --- | --- | --- | --- | --- |
| 1. Visiting friends or family members in their homes | 🞏 | 🞏 | 🞏 | 🞏 | 🞏 | 🞏 | 🞏 🞏 |
| 1. Going to social events, parties, or celebrations | 🞏 | 🞏 | 🞏 | 🞏 | 🞏 | 🞏 | 🞏 🞏 |
| 1. Having friends and family members visit you in your home | 🞏 | 🞏 | 🞏 | 🞏 | 🞏 | 🞏 | 🞏 🞏 |
| 1. Walking or getting around INSIDE your home | 🞏 | 🞏 | 🞏 | 🞏 | 🞏 | 🞏 | 🞏 🞏 |
| 1. Walking OUTSIDE, just to get around, in the area around your home or other places you need to go on a regular basis (This does not include walking for exercise) | 🞏 | 🞏 | 🞏 | 🞏 | 🞏 | 🞏 | 🞏 🞏 |
| 1. Taking part in leisure activities IN YOUR HOME, such as reading, watching television, listening to music | 🞏 | 🞏 | 🞏 | 🞏 | 🞏 | 🞏 | 🞏 🞏 |
| 1. Taking part in leisure activities OUTSIDE your home, such as **going to the pub**, bingo, going to **the cinema**, club meetings, restaurants | 🞏 | 🞏 | 🞏 | 🞏 | 🞏 | 🞏 | 🞏 🞏 |
| 1. Working on hobbies, crafts, or creative activities, such as music, knitting, sewing, woodworking, or painting | 🞏 | 🞏 | 🞏 | 🞏 | 🞏 | 🞏 | 🞏 🞏 |
| 1. Taking part in physical recreational activities, such as walking for exercise, dancing, playing golf, bicycling, swimming or water aerobics | 🞏 | 🞏 | 🞏 | 🞏 | 🞏 | 🞏 | 🞏 🞏 |

Because of your arthritis how much difficulty have you had with each of the following activities over the past week?

|  | **Does not apply to me** | **No difficulty** | **Some difficulty** | **Much difficulty** | **Unable to do** | **Not important  to me** | **Do you have to make**  **changes to how you do this activity because of your arthritis?**  **Yes No** |
| --- | --- | --- | --- | --- | --- | --- | --- |
| 1. Driving or getting around your community by public transport | 🞏 | 🞏 | 🞏 | 🞏 | 🞏 | 🞏 | 🞏 🞏 |
| 1. Travelling long distances | 🞏 | 🞏 | 🞏 | 🞏 | 🞏 | 🞏 | 🞏 🞏 |
| 1. Taking part in religious or spiritual activities or religious services | 🞏 | 🞏 | 🞏 | 🞏 | 🞏 | 🞏 | 🞏 🞏 |
| 1. Doing volunteer work | 🞏 | 🞏 | 🞏 | 🞏 | 🞏 | 🞏 | 🞏 🞏 |
| 1. Working at a job for pay | 🞏 | 🞏 | 🞏 | 🞏 | 🞏 | 🞏 | 🞏 🞏 |
| 1. Taking care of household business, e.g. pay bills or scheduling repairs | 🞏 | 🞏 | 🞏 | 🞏 | 🞏 | 🞏 | 🞏 🞏 |
| 1. Taking care of social communication such as writing letters, sending e-mails, making phone calls or texting | 🞏 | 🞏 | 🞏 | 🞏 | 🞏 | 🞏 | 🞏 🞏 |
| 1. Going to college or educational activities | 🞏 | 🞏 | 🞏 | 🞏 | 🞏 | 🞏 | 🞏 🞏 |
| 1. Sleeping | 🞏 | 🞏 | 🞏 | 🞏 | 🞏 | 🞏 | 🞏 🞏 |
| 1. Eating and chewing | 🞏 | 🞏 | 🞏 | 🞏 | 🞏 | 🞏 | 🞏 🞏 |
| 1. Meeting new people | 🞏 | 🞏 | 🞏 | 🞏 | 🞏 | 🞏 | 🞏 🞏 |
| 1. Having and/or taking care of a pet. | 🞏 | 🞏 | 🞏 | 🞏 | 🞏 | 🞏 | 🞏 🞏 |
| 1. Having intimate relations with your spouse/partner | 🞏 | 🞏 | 🞏 | 🞏 | 🞏 | 🞏 | 🞏 🞏 |

Because of your arthritis how much difficulty have you had with each of the following activities over the past week?

|  | **Does not apply to me** | **No difficulty** | **Some difficulty** | **Much difficulty** | **Unable to do** | **Not important  to me** | **Do you have to make**  **changes to how you do this activity because of your arthritis?**  **Yes No** |
| --- | --- | --- | --- | --- | --- | --- | --- |
| 1. Other activity? (please specify_________________________ | 🞏 | 🞏 | 🞏 | 🞏 | 🞏 | 🞏 | 🞏 🞏 |
| 1. Other activity? (please specify_________________________ | 🞏 | 🞏 | 🞏 | 🞏 | 🞏 | 🞏 | 🞏 🞏 |
| 1. Other activity? (please specify_________________________ | 🞏 | 🞏 | 🞏 | 🞏 | 🞏 | 🞏 | 🞏 🞏 |
